# Supplementary material for: A highly divergent cryptic SARS-CoV-2 lineage exhibits strong receptor binding and immune evasion
Source: medRxiv. 2025 Nov 20:2025.11.18.25340485. Preprint. [Version 1] doi: 10.1101/2025.11.18.25340485 (PMC12668080; doi:10.1101/2025.11.18.25340485)
Supplement: Supplement 1 [file media-1.pdf]

Extended Data Table 1. X-ray data collection and refinement statistics

**Data collection**	NJ-RBD + hACE2	NJ-RBD + CC25.4
Beamline	SSRL BL 12-1	SSRL BL 12-1
Wavelength (Å)	0.97946	0.97946
Space group	P 1 2_1 1	I 2
Unit cell parameters		
a, b, c (Å)	81.6, 123.0, 110.4	126.4, 239.5, 152.2
α, β, γ (°)	90, 91.1, 90	90, 113.9, 90
Resolution (Å) [a]	50.0-2.50 (2.54-2.50)	50.0-2.80 (2.85-2.80)
Unique reflections [a]	73,735 (3,725)	96,329 (4,834)
Redundancy [a]	6.9 (6.6)	6.9 (6.4)
Completeness (%) [a]	98.1 (98.8)	98.5 (99.2)
$<I/\sigma_I>$ [a]	17.0 (1.1)	14.5 (1.5)
R_{sym} [b] (%) [a]	11.7 (>100)	20.0 (>100)
R_{pim} [b] (%) [a]	4.7 (86.1)	8.1 (94.4)
$CC_{1/2}$ [c] (%) [a]	99.1 (49.7)	99.0 (63.3)
**Refinement statistics**		
Resolution (Å)	36.7-2.50	38.7-2.80
Reflections (work)	61,736	85,600
Reflections (test)	1,992	2,000
R_{cryst} [d] / R_{free} [e] (%)	22.0/26.6	22.5/25.9
Copies of complex per ASU	2	3
No. of atoms	13,349	18,171
Macromolecules	13,232	17,979
Ligands [f]	32	123
Waters	85	69
Average *B*-values (Å^2)	44	57
Macromolecules	44	57
Ligands [f]	70	78
Waters	30	38
Wilson *B*-value (Å^2)	37	53
**RMSD from ideal geometry**		
Bond length (Å)	0.002	0.002
Bond angle (°)	0.53	0.56
**Ramachandran statistics (%)** [g]		
Favored	97.3	96.2
Outliers	0.1	0.1
**PDB code**	9Z1F	9Z1G

[a] Numbers in parentheses refer to the highest resolution shell.

[b] $R_{sym} = \Sigma_{hkl} \Sigma_i | I_{hkl,i} - <I_{hkl}> | / \Sigma_{hkl} \Sigma_i I_{hkl,i}$ and $R_{pim} = \Sigma_{hkl} (1/(n-1))^{1/2} \Sigma_i | I_{hkl,i} - <I_{hkl}> | / \Sigma_{hkl} \Sigma_i I_{hkl,i}$, where $I_{hkl,i}$ is the scaled intensity of the ith measurement of reflection h, k, l, $<I_{hkl}>$ is the average intensity for that reflection, and *n* is the redundancy.

[c] $CC_{1/2}$ = Pearson correlation coefficient between two random half datasets.

[d] $R_{cryst} = \Sigma_{hkl} | F_o - F_c | / \Sigma_{hkl} | F_o |$ x 100, where F_o and F_c are the observed and calculated structure factors, respectively.

[e] R_{free} was calculated as for R_{cryst}, but on a test set comprising ~2.4%-5.1% of the data excluded from refinement.

[f] Bound ligands are ethylene glycol, zinc ion, and sulfate molecules.

[g] From MolProbity[1].

Extended Data Table 2. Sequences of the forward primers for constructing NJ-RBM mutant library.

Primer ID	Sequence (5' to 3')
Cassette1_1	ACCGGTTGTGTCATTGCATGGNNKTCTAAGACGCTTGACAGCAATAATAAGGGCAATACC
Cassette1_2	ACCGGTTGTGTCATTGCATGGAATNNKAAAACCCTTGACAGCAATAATAAGGGCAATACC
Cassette1_3	ACCGGTTGTGTCATTGCATGGAACTCTNNKACCTTAGACAGCAATAATAAGGGCAATACC
Cassette1_4	ACCGGTTGTGTCATTGCATGGAATAGTAAGNNKTTAGACAGCAATAATAAGGGCAATACC
Cassette1_5	ACCGGTTGTGTCATTGCATGGAACTCTAAAACGNNKGATAGCAATAATAAGGGCAATACC
Cassette1_6	ACCGGTTGTGTCATTGCATGGAACAGTAAGACCCTTNNKAGCAATAATAAGGGCAATACC
Cassette1_7	ACCGGTTGTGTCATTGCATGGAATTCTAAAACGTTAGACNNKAATAATAAGGGCAATACC
Cassette1_8	ACCGGTTGTGTCATTGCATGGAACAGTAAGACGTTAGATAGCNNKAATAAGGGCAATACC
Cassette2_1	AGTAAAACGCTTGACAGCAATNNKAAAGGTAATACCAAGTACAAGTTCCGTTTCGTGAGG
Cassette2_2	AGTAAAACGCTTGACAGCAATAACNNKGGCAACACCAAGTACAAGTTCCGTTTCGTGAGG
Cassette2_3	AGTAAAACGCTTGACAGCAATAACAAGNNKAATACAAAGTACAAGTTCCGTTTCGTGAGG
Cassette2_4	AGTAAAACGCTTGACAGCAATAATAAAGGCNNKACAAAGTACAAGTTCCGTTTCGTGAGG
Cassette2_5	AGTAAAACGCTTGACAGCAATAATAAGGGTAACNNKAAGTACAAGTTCCGTTTCGTGAGG
Cassette2_6	AGTAAAACGCTTGACAGCAATAACAAAGGTAACACANNKTACAAGTTCCGTTTCGTGAGG
Cassette2_7	AGTAAAACGCTTGACAGCAATAACAAGGGTAACACCAAANNKAAGTTCCGTTTCGTGAGG
Cassette2_8	AGTAAAACGCTTGACAGCAATAACAAGGGCAACACAAAATACNNKTTCCGTTTCGTGAGG
Cassette3_1	AAGGGCAATACCAAGTACAAGNNKCGATTTGTGAGGAAGAGCCGCTTGCAGCCCTTTGAA
Cassette3_2	AAGGGCAATACCAAGTACAAGTTTNNKTTCGTAAGGAAGAGCCGCTTGCAGCCCTTTGAA
Cassette3_3	AAGGGCAATACCAAGTACAAGTTTCGTNNKGTGCGAAAGAGCCGCTTGCAGCCCTTTGAA
Cassette3_4	AAGGGCAATACCAAGTACAAGTTCCGATTCNNKCGAAAGAGCCGCTTGCAGCCCTTTGAA
Cassette3_5	AAGGGCAATACCAAGTACAAGTTCCGTTTTGTANNKAAGAGCCGCTTGCAGCCCTTTGAA
Cassette3_6	AAGGGCAATACCAAGTACAAGTTTCGATTTGTACGANNKAGCCGCTTGCAGCCCTTTGAA
Cassette3_7	AAGGGCAATACCAAGTACAAGTTTCGATTCGTGCGAAAANNKCGCTTGCAGCCCTTTGAA
Cassette3_8	AAGGGCAATACCAAGTACAAGTTTCGTTTTGTAAGGAAAAGCNNKTTGCAGCCCTTTGAA
Cassette4_1	CGTTTCGTGAGGAAGAGCCGCNNKCAACCTTTTGAAAGGGACATCTCAACTGAAATTTTT
Cassette4_2	CGTTTCGTGAGGAAGAGCCGCTTANNKCCCTTCGAAAGGGACATCTCAACTGAAATTTTT
Cassette4_3	CGTTTCGTGAGGAAGAGCCGCTTACAGNNKTTTGAGAGGGACATCTCAACTGAAATTTTT
Cassette4_4	CGTTTCGTGAGGAAGAGCCGCTTGCAACCCNNKGAGAGGGACATCTCAACTGAAATTTTT
Cassette4_5	CGTTTCGTGAGGAAGAGCCGCTTGCAGCCTTTCNNKAGGGACATCTCAACTGAAATTTTT
Cassette4_6	CGTTTCGTGAGGAAGAGCCGCTTACAACCTTTCGAGNNKGACATCTCAACTGAAATTTTT
Cassette4_7	CGTTTCGTGAGGAAGAGCCGCTTACAACCCTTTGAGCGANNKATCTCAACTGAAATTTTT
Cassette4_8	CGTTTCGTGAGGAAGAGCCGCTTACAGCCTTTTGAACGAGACNNKTCAACTGAAATTTTT
Cassette5_1	CAGCCCTTTGAAAGGGACATCNNKACCGAGATTTTTCAAGCGGGTAATCGCCCATGCAAT
Cassette5_2	CAGCCCTTTGAAAGGGACATCTCTNNKGAAATCTTTCAAGCGGGTAATCGCCCATGCAAT
Cassette5_3	CAGCCCTTTGAAAGGGACATCTCTACTNNKATTTTCCAAGCGGGTAATCGCCCATGCAAT
Cassette5_4	CAGCCCTTTGAAAGGGACATCTCAACCGAANNKTTCCAAGCGGGTAATCGCCCATGCAAT
Cassette5_5	CAGCCCTTTGAAAGGGACATCTCAACTGAGATCNNKCAAGCGGGTAATCGCCCATGCAAT
Cassette5_6	CAGCCCTTTGAAAGGGACATCTCTACCGAGATCTTCNNKGCGGGTAATCGCCCATGCAAT
Cassette5_7	CAGCCCTTTGAAAGGGACATCTCTACCGAAATTTTTCAGNNKGGTAATCGCCCATGCAAT
Cassette5_8	CAGCCCTTTGAAAGGGACATCTCTACTGAGATCTTTCAGGCGNNKAATCGCCCATGCAAT
Cassette6_1	ACTGAAATTTTTCAAGCGGGTNNKCGACCTTGCAATACCGTGGGCCTTAATTGCTACCAC
Cassette6_2	ACTGAAATTTTTCAAGCGGGTAACNNKCCATGTAATACCGTGGGCCTTAATTGCTACCAC
Cassette6_3	ACTGAAATTTTTCAAGCGGGTAACCGCNNKTGCAACACCGTGGGCCTTAATTGCTACCAC
Cassette6_4	ACTGAAATTTTTCAAGCGGGTAATCGACCANNKAACACCGTGGGCCTTAATTGCTACCAC
Cassette6_5	ACTGAAATTTTTCAAGCGGGTAATCGCCCTTGTNNKACCGTGGGCCTTAATTGCTACCAC
Cassette6_6	ACTGAAATTTTTCAAGCGGGTAACCGACCTTGTAACNNKGTGGGCCTTAATTGCTACCAC
Cassette6_7	ACTGAAATTTTTCAAGCGGGTAACCGACCATGCAACACANNKGGCCTTAATTGCTACCAC
Cassette6_8	ACTGAAATTTTTCAAGCGGGTAACCGCCCTTGTAATACAGTGNNKCTTAATTGCTACCAC
Cassette7_1	CGCCCATGCAATACCGTGGGCNNKAACTGTTACCACCCCTTGCTGACATACAACTTTCAA
Cassette7_2	CGCCCATGCAATACCGTGGGCTTANNKTGCTATCACCCCTTGCTGACATACAACTTTCAA
Cassette7_3	CGCCCATGCAATACCGTGGGCTTAAATNNKTACCATCCCTTGCTGACATACAACTTTCAA
Cassette7_4	CGCCCATGCAATACCGTGGGCCTTAACTGCNNKCATCCCTTGCTGACATACAACTTTCAA
Cassette7_5	CGCCCATGCAATACCGTGGGCCTTAATTGTTATNNKCCCTTGCTGACATACAACTTTCAA
Cassette7_6	CGCCCATGCAATACCGTGGGCTTAAACTGTTATCATNNKTTGCTGACATACAACTTTCAA
Cassette7_7	CGCCCATGCAATACCGTGGGCTTAAACTGCTACCATCCTNNKCTGACATACAACTTTCAA
Cassette7_8	CGCCCATGCAATACCGTGGGCTTAAATTGTTATCACCCTTTGNNKACATACAACTTTCAA
Cassette8_1	AATTGCTACCACCCCTTGCTGNNKTATAATTTTCAACCCACTAGTGGTGTCGGTCATCAA
Cassette8_2	AATTGCTACCACCCCTTGCTGACCNNKAACTTCCAACCCACTAGTGGTGTCGGTCATCAA
Cassette8_3	AATTGCTACCACCCCTTGCTGACCTACNNKTTTCAGCCCACTAGTGGTGTCGGTCATCAA
Cassette8_4	AATTGCTACCACCCCTTGCTGACATATAACNNKCAGCCCACTAGTGGTGTCGGTCATCAA
Cassette8_5	AATTGCTACCACCCCTTGCTGACATACAATTTCNNKCCCACTAGTGGTGTCGGTCATCAA
Cassette8_6	AATTGCTACCACCCCTTGCTGACCTATAATTTCCAGNNKACTAGTGGTGTCGGTCATCAA
Cassette8_7	AATTGCTACCACCCCTTGCTGACCTATAACTTTCAACCTNNKAGTGGTGTCGGTCATCAA
Cassette8_8	AATTGCTACCACCCCTTGCTGACCTACAACTTCCAGCCTACTNNKGGTGTCGGTCATCAA
Cassette9_1	TACAACTTTCAACCCACTAGTNNKGTAGGGCATCAACCGCATAGAGTGGTGGTTCTC
Cassette9_2	TACAACTTTCAACCCACTAGTGGGNNKGGTCACCAACCGCATAGAGTGGTGGTTCTC
Cassette9_3	TACAACTTTCAACCCACTAGTGGTGTANNKCACCAGCCGCATAGAGTGGTGGTTCTC
Cassette9_4	TACAACTTTCAACCCACTAGTGGGGTCGGGNNKCAGCCGCATAGAGTGGTGGTTCTC
Cassette9_5	TACAACTTTCAACCCACTAGTGGTGTAGGTCATNNKCCTCATAGAGTGGTGGTTCTC
Cassette9_6	TACAACTTTCAACCCACTAGTGGTGTCGGGCACCAANNKCATAGAGTGGTGGTTCTC
Cassette9_7	TACAACTTTCAACCCACTAGTGGGGTAGGTCATCAGCCGNNKAGAGTGGTGGTTCTC

Extended Data Table 3. Sequences of the reverse primers for constructing NJ-RBM mutant library.

Primer ID	Sequence (5' to 3')
Cassette1_R	CCATGCAATGACACAACCGGT
Cassette2_R	ATTGCTGTCAAGCGTTTTACT
Cassette3_R	CTTGTACTTGGTATTGCCCTT
Cassette4_R	GCGGCTCTTCCTCACGAAACG
Cassette5_R	GATGTCCCTTTCAAAGGGCTG
Cassette6_R	ACCCGCTTGAAAAATTTCAGT
Cassette7_R	GCCCACGGTATTGCATGGGCG
Cassette8_R	CAGCAAGGGGTGGTAGCAATT
Cassette9_R	ACTAGTGGGTTGAAAGTTGTA

```
Positions: 334 346 367 369 372 384
NJ-RBD                  TKLCPFGEVFNATNFASVYAWNRKRISNCVADYSFLSNS-SFSTFKCYGVSHTKLNDLCF
WT-RBD                  TNLCPFGEVFNATRFASVYAWNRKRISNCVADYSVLYNSASFSTFKCYGVSPTKLNDLCF
Alpha-RBD               TNLCPFGEVFNATRFASVYAWNRKRISNCVADYSVLYNSASFSTFKCYGVSPTKLNDLCF
Beta-RBD                TNLCPFGEVFNATRFASVYAWNRKRISNCVADYSVLYNSASFSTFKCYGVSPTKLNDLCF
Delta-RBD               TNLCPFGEVFNATRFASVYAWNRKRISNCVADYSVLYNSASFSTFKCYGVSPTKLNDLCF
Omicron_BA.1-RBD        TNLCPFDEVFNATKFASVYAWNRKRISNCVADYSVLYNLAPFFTFKCYGVSPTKLNDLCF
Omicron_BA.2-RBD        TNLCPFDEVFNATRFASVYAWNRKRISNCVADYSVLYNFAPFFAFKCYGVSPTKLNDLCF
Omicron_BA.4/5-RBD      TNLCPFDEVFNATRFASVYAWNRKRISNCVADYSVLYNFAPFFAFKCYGVSPTKLNDLCF
Omicron_BQ.1.1-RBD      TNLCPFDEVFNATTFASVYAWNRKRISNCVADYSVLYNFAPFFAFKCYGVSPTKLNDLCF
Omicron_XBB.1.5-RBD     TNLCPFHEVFNATTFASVYAWNRKRISNCVADYSVIYNFAPFFAFKCYGVSPTKLNDLCF
Omicron_BA.2.86-RBD     TNLCPFHEVFNATRFASVYAWNRTRISNCVADYSVLYNFAPFFAFKCYGVSPTKLNDLCF
Omicron_JN.1-RBD        TNLCPFHEVFNATRFASVYAWNRTRISNCVADYSVLYNFAPFFAFKCYGVSPTKLNDLCF
Omicron_KP.2-RBD        TNLCPFHEVFNATTFASVYAWNRTRISNCVADYSVLYNFAPFFAFKCYGVSPTKLNDLCF
Omicron_KP.3-RBD        TNLCPFHEVFNATRFASVYAWNRTRISNCVADYSVLYNFAPFFAFKCYGVSPTKLNDLCF
                        *:**** ****** *********.**********.: *   * :******* ********

Positions: 403 408 413 415 421 439 440 444 445 446 449 450 452
NJ-RBD                  TNVYADSFVIKGDEVTQIAPRQEGKIADFNYKLPDDFTGCVIAWNSKTLDSNNKGNTKYK
WT-RBD                  TNVYADSFVIRGDEVRQIAPGQTGKIADYNYKLPDDFTGCVIAWNSNNLDSKVGGNYNYL
Alpha-RBD               TNVYADSFVIRGDEVRQIAPGQTGKIADYNYKLPDDFTGCVIAWNSNNLDSKVGGNYNYL
Beta-RBD                TNVYADSFVIRGDEVRQIAPGQTGNIADYNYKLPDDFTGCVIAWNSNNLDSKVGGNYNYL
Delta-RBD               TNVYADSFVIRGDEVRQIAPGQTGKIADYNYKLPDDFTGCVIAWNSNNLDSKVGGNYNYR
Omicron_BA.1-RBD        TNVYADSFVIRGDEVRQIAPGQTGNIADYNYKLPDDFTGCVIAWNSNKLDSKVSGNYNYL
Omicron_BA.2-RBD        TNVYADSFVIRGNEVSQIAPGQTGNIADYNYKLPDDFTGCVIAWNSNKLDSKVGGNYNYL
Omicron_BA.4/5-RBD      TNVYADSFVIRGNEVSQIAPGQTGNIADYNYKLPDDFTGCVIAWNSNKLDSKVGGNYNYR
Omicron_BQ.1.1-RBD      TNVYADSFVIRGNEVSQIAPGQTGNIADYNYKLPDDFTGCVIAWNSNKLDSTVGGNYNYR
Omicron_XBB.1.5-RBD     TNVYADSFVIRGNEVSQIAPGQTGNIADYNYKLPDDFTGCVIAWNSNKLDSKPSGNYNYL
Omicron_BA.2.86-RBD     TNVYADSFVIKGNEVSQIAPGQTGNIADYNYKLPDDFTGCVIAWNSNKLDSKHSGNYDYW
Omicron_JN.1-RBD        TNVYADSFVIKGNEVSQIAPGQTGNIADYNYKLPDDFTGCVIAWNSNKLDSKHSGNYDYW
Omicron_KP.2-RBD        TNVYADSFVIKGNEVSQIAPGQTGNIADYNYKLPDDFTGCVIAWNSNKLDSKHSGNYDYW
Omicron_KP.3-RBD        TNVYADSFVIKGNEVSQIAPGQTGNIADYNYKLPDDFTGCVIAWNSNKLDSKHSGNYDYW
                        **********:*:** **** * *:***:*****************:.***.  ** .*

Positions: 453 455 456 460 462 473 477 478 482 483 484 486 490 493 496 501 505 508
NJ-RBD                  FRFVRKSRLQPFERDISTEIFQAGNRPCNT-VGLNCYHPLLTYNFQPTSGVGHQPHRVVV
WT-RBD                  YRLFRKSNLKPFERDISTEIYQAGSTPCNGVEGFNCYFPLQSYGFQPTNGVGYQPYRVVV
Alpha-RBD               YRLFRKSNLKPFERDISTEIYQAGSTPCNGVEGFNCYFPLQSYGFQPTYGVGYQPYRVVV
Beta-RBD                YRLFRKSNLKPFERDISTEIYQAGSTPCNGVKGFNCYFPLQSYGFQPTYGVGYQPYRVVV
Delta-RBD               YRLFRKSNLKPFERDISTEIYQAGSKPCNGVEGFNCYFPLQSYGFQPTNGVGYQPYRVVV
Omicron_BA.1-RBD        YRLFRKSNLKPFERDISTEIYQAGNKPCNGVAGFNCYFPLRSYSFRPTYGVGHQPYRVVV
Omicron_BA.2-RBD        YRLFRKSNLKPFERDISTEIYQAGNKPCNGVAGFNCYFPLRSYGFRPTYGVGHQPYRVVV
Omicron_BA.4/5-RBD      YRLFRKSNLKPFERDISTEIYQAGNKPCNGVAGVNCYFPLQSYGFRPTYGVGHQPYRVVV
Omicron_BQ.1.1-RBD      YRLFRKSKLKPFERDISTEIYQAGNKPCNGVAGVNCYFPLQSYGFRPTYGVGHQPYRVVV
Omicron_XBB.1.5-RBD     YRLFRKSKLKPFERDISTEIYQAGNKPCNGVAGPNCYSPLQSYGFRPTYGVGHQPYRVVV
Omicron_BA.2.86-RBD     YRLFRKSKLKPFERDISTEIYQAGNKPCKG-KGPNCYFPLQSYGFRPTYGVGHQPYRVVV
Omicron_JN.1-RBD        YRSFRKSKLKPFERDISTEIYQAGNKPCKG-KGPNCYFPLQSYGFRPTYGVGHQPYRVVV
Omicron_KP.2-RBD        YRSLRKSKLKPFERDISTEIYQAGNKPCKG-KGPNCYFPLQSYGFRPTYGVGHQPYRVVV
Omicron_KP.3-RBD        YRSLRKSKLKPFERDISTEIYQAGNKPCKG-KGPNCYFPLESYGFRPTYGVGHQPYRVVV
                        :* .***.*:**********:***. **:   * *** ** :*.*:** ***:**:****

Positions: 519 529
NJ-RBD                  LSFELLQAPATVCGPKRS
WT-RBD                  LSFELLHAPATVCGPKKS
Alpha-RBD               LSFELLHAPATVCGPKKS
Beta-RBD                LSFELLHAPATVCGPKKS
Delta-RBD               LSFELLHAPATVCGPKKS
Omicron_BA.1-RBD        LSFELLHAPATVCGPKKS
Omicron_BA.2-RBD        LSFELLHAPATVCGPKKS
Omicron_BA.4/5-RBD      LSFELLHAPATVCGPKKS
Omicron_BQ.1.1-RBD      LSFELLHAPATVCGPKKS
Omicron_XBB.1.5-RBD     LSFELLHAPATVCGPKKS
Omicron_BA.2.86-RBD     LSFELLHAPATVCGPKKS
Omicron_JN.1-RBD        LSFELLHAPATVCGPKKS
Omicron_KP.2-RBD        LSFELLHAPATVCGPKKS
Omicron_KP.3-RBD        LSFELLHAPATVCGPKKS
                        ******:*********:*
```

Extended Data Fig 1. Sequence alignment of RBDs including cryptic lineage NJ and several VOC/VOIs. Red indicates unique substitutions in NJ-RBD. Blue indicates convergent substitutions between NJ-RBD and other VOC/VOI RBDs. Residues identical in all aligned sequences are labeled by an asterisk (*), whereas a colon (:) and a period (.) indicate highly similar and less similar sequences, respectively. The sequence alignment was performed using Clustal Omega[2].

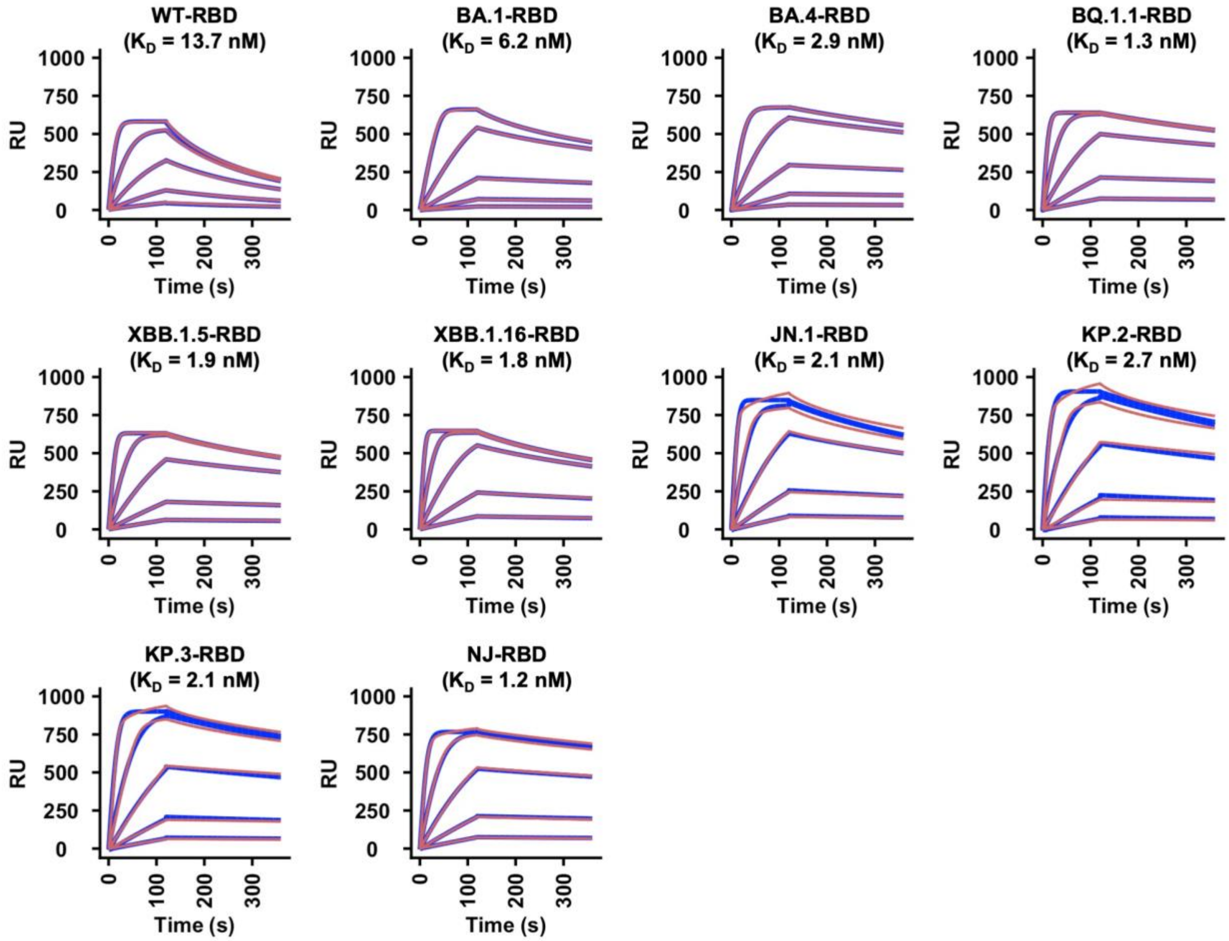

Extended Data Fig 2. Receptor binding of various RBDs to hACE2 using SPR. Biacore sensorgram representing the binding of the indicated RBD to hACE2. The y-axis shows the response unit (RU). Red lines represent the response curve, and blue lines represent a 1:1 binding model. Binding kinetics were measured against hACE2 at 0 nM, 3.7 nM, 11.1 nM, 33.3 nM, 100 nM, and 300 nM. Dissociation constant (K_D) is indicated.

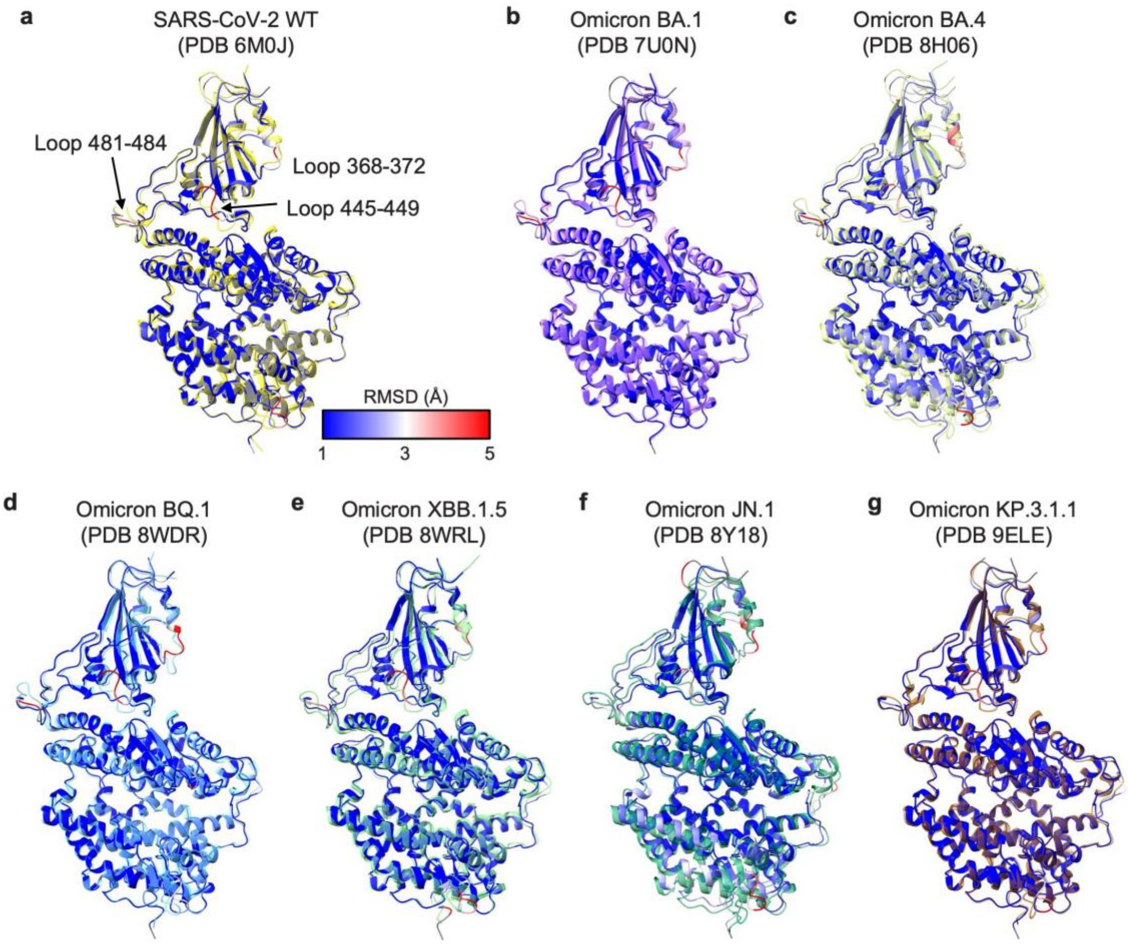

Extended Data Fig 3. Structural comparison between hACE2 in complex with NJ-RBD and in complex with RBDs from other VOCs/VOIs. Positions of loops 368-372, 445-449, and 481-484 are labeled. Structural alignment of the NJ-RBD/hACE2 structure (this study, blue) to hACE2 in complex with **(a)** SARS-CoV-2 WT (PDB 6M0J, yellow)[3], **(b)** Omicron BA.1 (PDB 7U0N, pink)[4], **(c)** BA.4 (PDB 8H06, lime)[5], **(d)** BQ.1 (PDB 8WDR, cyan)[6], **(e)** XBB.1.5 (PDB 8WRL, light green)[7], **(f)** JN.1 (PDB 8Y18, green)[8], and **(g)** KP.3.1.1 (PDB 9ELE, brown)[9], respectively. Structural differences of NJ-RBD/hACE2 are color-coded by their root mean square deviation (RMSD) (Å).

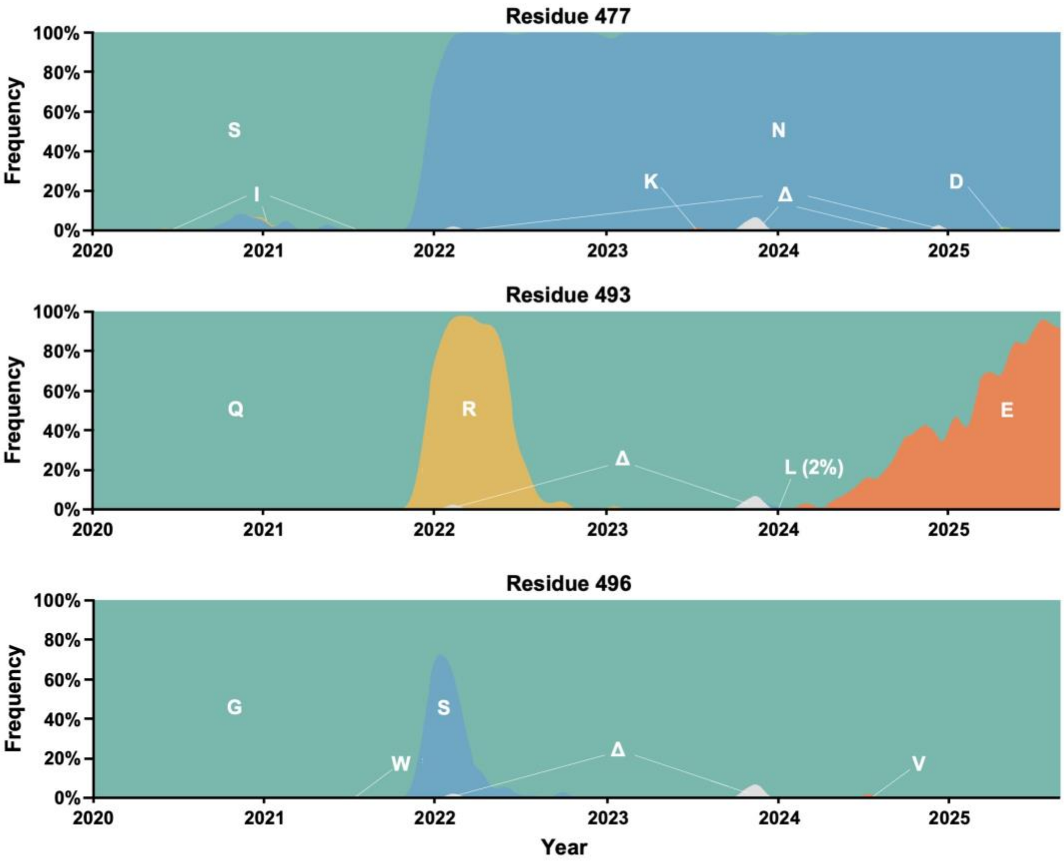

Extended Data Fig 4. Frequency of amino acids at positions 477, 493, and 496 of SARS-CoV-2 spike as of July 2025. Plot was generated using Nextstrain (nextstrain.org)[10], which sources data from GISAID[11]. "Δ" indicates deletion.

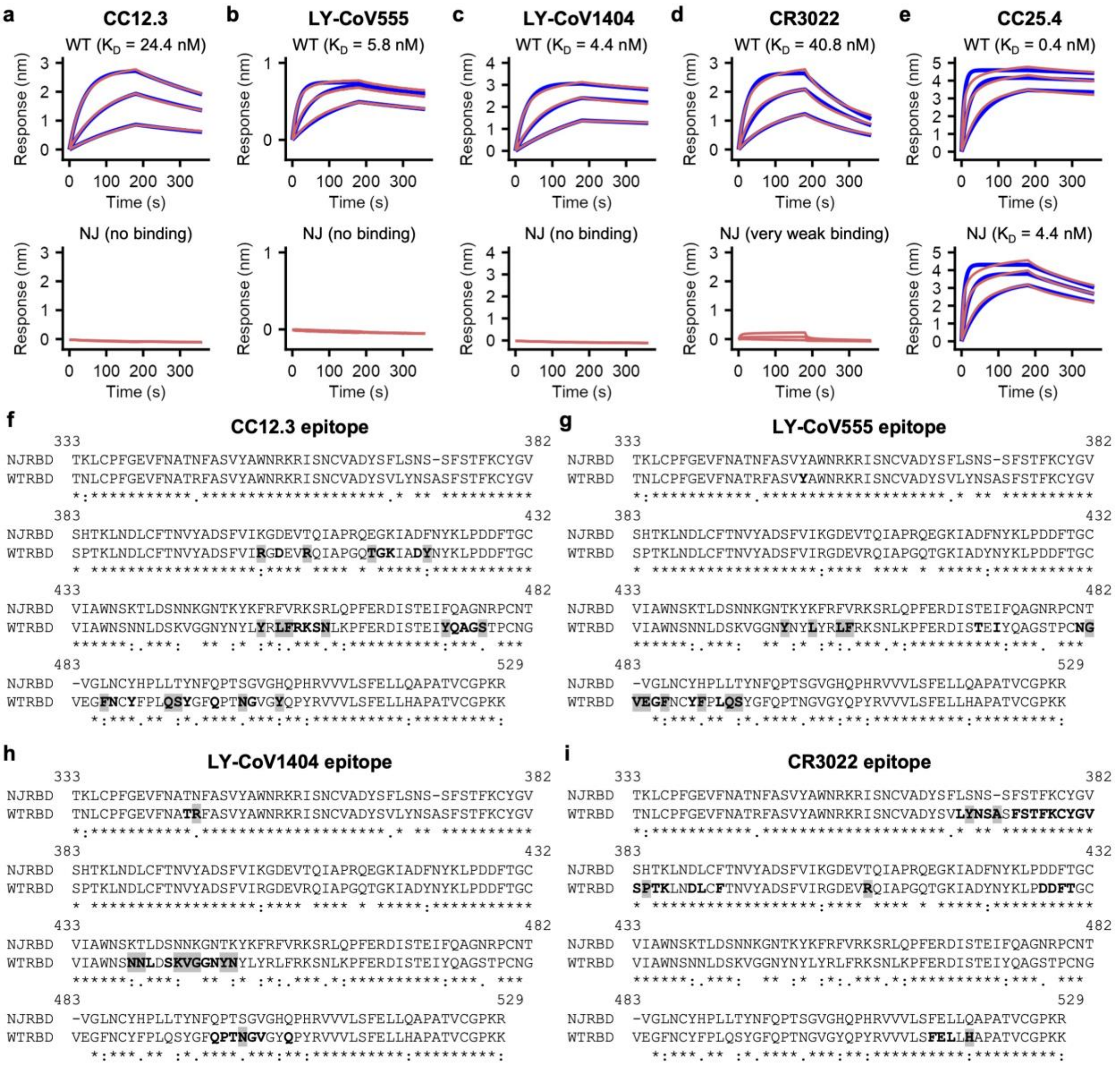

Extended Data Fig 5. Sensorgrams for binding of WT-RBD and NJ-RBD to neutralizing antibodies. Binding kinetics of WT-RBD and NJ-RBD against **(a)** CC12.3, **(b)** LY-CoV555, **(c)** LY-CoV1404, **(d)** CR3022, and **(e)** CC25.4 Fabs were measured by biolayer interferometry (BLI). Red lines represent the response curve. Blue lines represent a 1:1 binding model. Binding kinetics were measured for antibody concentrations at 33.3 nM, 100 nM, and 300 nM. Dissociation constant (K_D) is indicated. **(f-i)** Sequence alignment of NJ-RBD and WT-RBD with the indicated epitope residues (BSA > 0 Å^2 as calculated by PISA[12]) in bold. Non-conserved epitope residues are highlighted in grey.

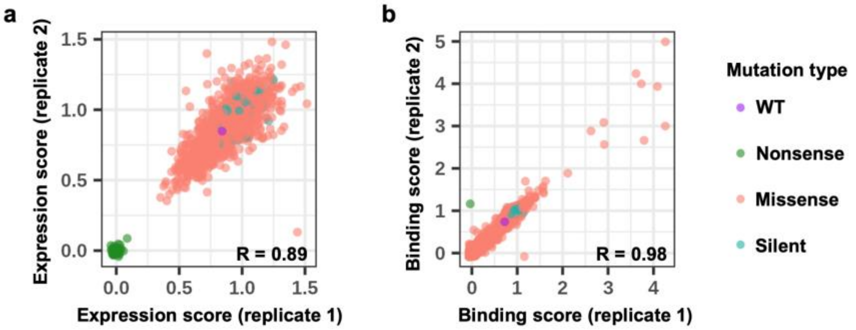

Extended Data Fig 6. Correlation between biological replicates of binding and expression sorts of NJ-RBM variant library. a, Correlation of expression scores between two independent biological replicates is shown. **b**, Correlation of binding scores between two independent biological replicates is shown. Mutation types are color-coded as follows: pink (missense), green (nonsense), blue (silent), and purple (WT). The Pearson correlation coefficient (R) is indicated. Please see Figure 5 for expression and binding analyses.

References

1 Chen, V. B. *et al.* MolProbity: all-atom structure validation for macromolecular crystallography. *Acta Crystallogr D Biol Crystallogr* **66**, 12-21 (2010). https://doi.org/10.1107/S0907444909042073

2 Sievers, F. *et al.* Fast, scalable generation of high-quality protein multiple sequence alignments using Clustal Omega. *Mol Syst Biol* **7**, 539 (2011). https://doi.org/10.1038/msb.2011.75

3 Lan, J. *et al.* Structure of the SARS-CoV-2 spike receptor-binding domain bound to the ACE2 receptor. *Nature* **581**, 215-220 (2020). https://doi.org/10.1038/s41586-020-2180-5

4 Geng, Q. *et al.* Structural basis for human receptor recognition by SARS-CoV-2 Omicron variant BA.1. *J Virol* **96**, e0024922 (2022). https://doi.org/10.1128/jvi.00249-22

5 Zhao, Z. *et al.* Structural basis for receptor binding and broader interspecies receptor recognition of currently circulating Omicron sub-variants. *Nat Commun* **14**, 4405 (2023). https://doi.org/10.1038/s41467-023-39942-z

6 Li, W. *et al.* Key mechanistic features of the trade-off between antibody escape and host cell binding in the SARS-CoV-2 Omicron variant spike proteins. *EMBO J* **43**, 1484-1498 (2024). https://doi.org/https://doi.org/10.1038/s44318-024-00062-z

7 Jian, F. *et al.* Convergent evolution of SARS-CoV-2 XBB lineages on receptor-binding domain 455–456 synergistically enhances antibody evasion and ACE2 binding. *PLoS Pathog* **19**, e1011868 (2023). https://doi.org/10.1371/journal.ppat.1011868

8 Li, L. *et al.* Spike structures, receptor binding, and immune escape of recently circulating SARS-CoV-2 Omicron BA.2.86, JN.1, EG.5, EG.5.1, and HV.1 sub-variants. *Structure* **32**, 1055-1067.e6 (2024). https://doi.org/https://doi.org/10.1016/j.str.2024.06.012

9 Feng, Z. *et al.* Structural and functional insights into the evolution of SARS-CoV-2 KP.3.1.1 spike protein. *Cell Rep* **44**, 115941 (2025). https://doi.org/10.1016/j.celrep.2025.115941

10 Hadfield, J. *et al.* Nextstrain: real-time tracking of pathogen evolution. *Bioinformatics* **34**, 4121-4123 (2018). https://doi.org/10.1093/bioinformatics/bty407

11 Shu, Y. & McCauley, J. GISAID: Global initiative on sharing all influenza data - from vision to reality. *Euro Surveill* **22**, 30494 (2017). https://doi.org/10.2807/1560-7917.Es.2017.22.13.30494

12 Krissinel, E. & Henrick, K. Inference of macromolecular assemblies from crystalline state. *J Mol Biol* **372**, 774-797 (2007). https://doi.org/10.1016/j.jmb.2007.05.022
